# Comparison of radiological changes after single- position versus dual- position for lateral interbody fusion and pedicle screw fixation

**DOI:** 10.1186/s12891-019-2992-3

**Published:** 2019-12-12

**Authors:** Akihiko Hiyama, Hiroyuki Katoh, Daisuke Sakai, Masato Sato, Masahiro Tanaka, Masahiko Watanabe

**Affiliations:** Department of Orthopaedic Surgery, Surgical Science, Tokai University School of Medicine, 143 Shimokasuya, Isehara, Kanagawa 259-1193 Japan

**Keywords:** Lateral lumbar interbody fusion (LLIF), single-position (SP), dual-position surgery (DP)

## Abstract

**Background:**

There have been few comparisons between dual positions, which require a position change, and a single position, which does not require position change, and it is not clear whether there is a difference in indirect decompression achieved by the two procedures. Therefore, the purpose of this study was to compare perioperative and radiographic outcomes following lateral lumbar interbody fusion (LLIF) in two cohorts of patients who underwent surgery in a single position or dual position.

**Methods:**

This study involved 45 patients who underwent indirect decompression at 68 levels, with LLIF and percutaneous pedicle screw (PPS) fixation for lumbar degenerative spondylolisthesis with spinal canal stenosis. Patient demographics and perioperative data were compared between two groups: patients who remained in the lateral decubitus position for pedicle screw fixation (SP group) and those turned to the prone position (DP group).

**Results:**

A total of 26 DP and 19 SP patients were analyzed. The operation time was approximately 31 min longer for the DP group (129.7 ± 36.0 min) than for the SP group (98.4 ± 41.3 min, *P* < 0.01). We also evaluated the pre- and postoperative image measurements, there was no significant difference for lumbar lordosis, segmental disc angle, slipping length, and disc height between the groups. The CSA of the dural sac (DP group, from 55.3 to 78.4 mm^2^; SP group, from 54.7 to 77.2 mm^2^) and central canal diameter (DP group, from 5.9 to 7.9 mm; SP group, from 5.6 to 7.7 mm) was significantly larger after surgery in both groups. However, there were no statistically significant differences between the two groups (*P* = 0.684).

**Conclusions:**

SP surgery could reduce the average surgery time by about 31 min. We found that the effect of indirect decompression by SP-PPS fixation following LLIF was considered to be a useful technique with no difference in dural sac enlargement or disc angle obtained compared with DP-PPS fixation.

## Background

There are several spinal surgery techniques that can be used to achieve either direct or indirect neural decompression [1]. Lateral lumbar interbody fusion (LLIF) is a minimally invasive surgical technique that allows access to the intervertebral disc space and vertebral bodies via a retroperitoneal transpsoas approach [2–4]. The advantage of LLIF over decompressive laminectomy is that it does not destroy the posterior elements and risk subsequent kyphosis. The benefits of this procedure also include reduced blood loss, reduced hospitalization, and reduced postoperative pain compared with open surgery. Numerous radiographic studies have shown significant improvements in foraminal height, posterior disc height (DH), and cross-sectional area (CSA) of the dural sac after indirect decompression [3–6].

In Japan, LLIF approaches have required repositioning the patient into the prone position for supplemental fixation with bilateral pedicle screws because of the higher rates of nonunion and subsidence after the LLIF procedure [7]. In the current procedure, after completion of the lateral access surgery, the patient is repositioned in the prone position for the percutaneous pedicle screw (PPS) fixation [8]. However, this repositioning requires a second round of preparation, draping, and room positioning, which increases the operation time and cost because of the extra use of materials.

Therefore, we considered that a technique of PPS insertion with the patient in the lateral decubitus position may be beneficial for treating patients who undergo LLIF in the lateral decubitus position. Previous study comparing lumbar spinal alignment after fusion by LLIF plus PPS fixation have reported that there were no differences in segmental lordosis and overall lordosis between patients treated using one position and two positions [9]. However, there have been few comparisons between dual positions, which require position change, and a single position, which does not require position change, and it is not clear whether there is a difference in indirect decompression achieved by the two procedures.

The purpose of this study was to compare perioperative and radiographic outcomes following LLIF (using extreme lateral interbody fusion [XLIF, NuVasive, Inc., CA, USA]) in two cohorts of patients who underwent either single-position (SP) or dual-position surgery (DP).

## Methods

### Included patients

For this study, we retrospectively reviewed the data of patients and met the following inclusion criteria. The inclusion criteria were patients aged > 18 years with lumbar spinal canal stenosis who underwent a combined operation (indirect decompression) using LLIF with lumbar degenerative spondylolisthesis (DS) at a single institute from January 2016 to July 2019. Basically, surgery by SP was started from January 2019. The method of surgery (SP or DP) was determined at the surgeon’s discretion.

All patients were diagnosed based on detailed history, neurological examinations, radiographic examination, myelograms, computed tomography (CT) after myelography, and/or magnetic resonance imaging (MRI). The indications were neurogenic claudication because of central or foraminal spinal stenosis. The conditions for diagnosis of spondylolisthesis and the inclusion criteria for fusion surgery were (1) more than 5% slip of the lumbar vertebra in a neutral position; or (2) more than 3-mm translation between flexion and extension positions on radiographic evaluation [10]. Stenosis location was recorded by the operating surgeon based on his evaluation of preoperative imaging studies. The exclusion criteria included patients who had undergone previous lumbar spinal surgery or those who were undergoing combined procedures including direct posterior decompression and posterior lumbar fusion.

The patient demographics and operative data (blood loss, operation time, and change in Hb level from before to the first day after surgery) were recorded. Length of stay and the intraoperative complication rate were also recorded for each patient. Imaging consisting of preoperative and postoperative radiological parameters and MRI was examined.

### Operative technique (XLIF and PPS fixation)

All patients underwent minimally invasive LLIF surgery utilizing the XLIF technique, which has been described previously [8, 11]. Briefly, the patient was placed in the lateral decubitus position with the hip at the level of the break in the operating table. The chest and hip areas were secured to the table with tape. Once the position was decided, the XLIF was performed as described previously. This facilitates access to the largest number of disc spaces with a relatively small incision. Blunt dissection was then used to access the disc spaces under fluoroscopic guidance. After removal of the disc material with a rongeur, a Cobb elevator was advanced gently under fluoroscopy guidance along the endplates to release the contralateral annulus. Cage-size trials were followed by additional disc curettage and rasping of the endplates. All cages were inserted using two containment sliders to protect the endplates and to keep the graft material inside the cage. For all patients, the side-to-side cage size was decided according to the width of the endplates at that level based on intraoperative fluoroscopic guidance, and titanium cages of a standard 18-mm width were used. The maximum distraction achieved during discectomy using the trial inserts provided guidance as to the height of the cage. The choice of these XLIF cages (CoRoent XL; NuVasive Inc.) was decided by the surgeon. Cage lengths ranged from 45 to 60 mm, and heights from 8 to 11 mm.

Following the XLIF, patients in the DP group were turned to the prone position and then re-prepared and re-draped. Bilateral PPS surgery was then performed with the patient in the prone position. Patients in the SP group remained in the lateral decubitus position for PPS fixation. In the SP group, most PPS procedures used the guidewire-less system VIPER PRIME™ (DePuy Synthes Spine, Raynham, MA, USA). An image in the anteroposterior view was taken to mark the lateral radiographic borders of the pedicles for screw placement. Using a lateral view, the centre of each pedicle was identified and marked [12]. A small incision 2–3 cm lateral to the lateral radiographic borders of each pedicle was made for percutaneous exposure and the stylets were then docked at the junction of the transverse process and the superior articular process. The stylets were then inserted with a hammer to hold the spot within the pedicles. After the stylets were inserted into the pedicle inner rim, an image in the AP view was taken to confirm from the lateral view that the posterior body wall had been reached. At that point, the C-arm was brought to a lateral position to maximize the working space for screw placement. After all screws had been inserted, a rod was passed percutaneously and secured to the screw heads using setscrews.

### Radiographical assessment

**Fig. 1:**
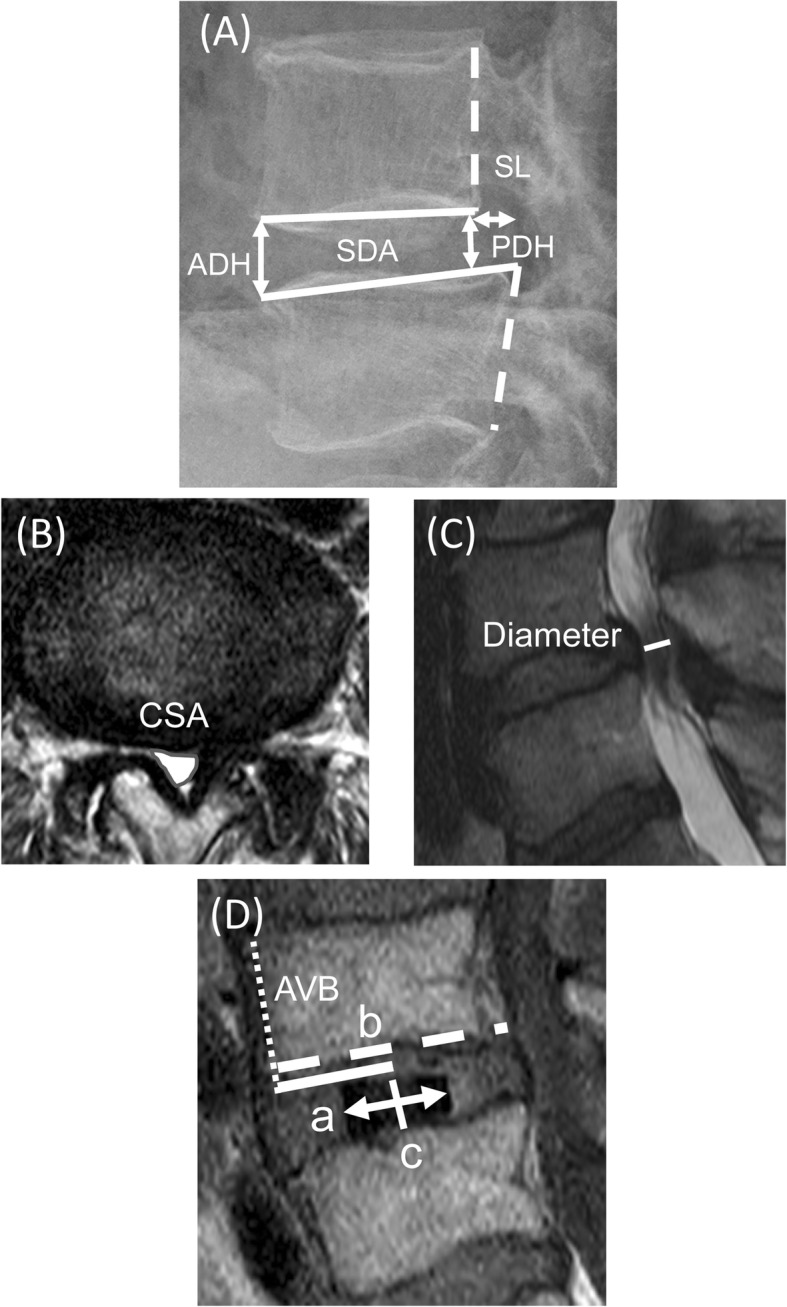
A radiographic analysis of XLIF cage positioning. (**A**) Plain lateral radiographs: segmental disc angle (SDA), disc height (DH), and slipping length (SL). (**B**) Magnetic resonance imaging (MRI)-T2 axial: cross-sectional spinal canal area (CSA). (**C**) MRI-T2 sagittal: diameter of the spinal canal. ADH; anterior disc height, PDH; posterior disc height. (**D**) The cage centre (c) is located by the midpoint between the anterior and posterior radiomarkers of the cage. The solid line (a) indicates distance between the anterior vertebral border (AVB) and the centre of the cage. The dotted line (b) indicates the anteroposterior width of the superior end plate. The cage positioning was measured using a Magnetic resonance imaging T1- weighted imaging and the value (a) divided by the full distance of the endplate (b) was expressed

X-ray evaluation involved examination of standing erect whole-spine antero-posterior and lateral full-spine radiographs. Radiographic assessment was performed using pre- and postoperative AP and lateral lumbar films to evaluate LL, SDA, SL, and DH (average anterior disc height [ADH], posterior disc heights [PDH], and average disc height [Av DH]). Av DH was defined as the average of anterior and posterior height determined from X-rays. MRI was also performed to determine cage placement (using the sagittal plane on T1-weighted imaging) and central canal dimensions (CSA and diameter using the axial and sagittal planes on T2-weighted imaging) [5, 13]. To determine placement of the XLIF cage, we measured the distance (a) between the anterior edge and the centre of the cage on the superior end plates, and expressed the value (a) divided by the full distance of the endplate (b) using a T1-weighted MRI (Fig. 1). X-ray and MRI were performed before surgery and at approximately 2 weeks and 2 months after surgery. We used a 1.5- or 3.0-T MRI system (Ingenia or Achieva; Philips Medical Systems, Best, the Netherlands) in this study. The average of image measurements determined by two examiners, including the authors, was used in analysis.

### Statistical analysis

Statistical analyses were performed using IBM SPSS Statistics version 20.0 (IBM Corp., Armonk, NY, USA). All values are expressed as mean ± standard deviation. Univariate differences between DP and SP groups were assessed using independent-sample *t* tests or the Mann–Whitney *U* test for data that were not normally distributed. The correlations between cage position and radiological parameters were analyzed using Spearman’s product-moment correlation coefficient.

For all statistical analyses, the type 1 error was set at 5% and *P* < 0.05 was considered to be significant.

## Results

A total of 45 patients (aged 71.4 ± 8.2 years; 29 men, 16 female) were included in the study and treated according to the protocol; 26 were classified in the DP group and 19 in the SP group. All patient characteristics and operative details are given in Table 1. Indirect decompression was performed at 68 levels (mean level: 1.5 ± 0.7), including 27 single-level cases, 14 two-level cases, 3 three-level cases, and one four-level case.

**Table 1:** Demographic and clinical summary

Characteristic	Statistic
No. of patients	45
Age (*years*)	71.4 ± 8.2
Female	16 (35.6%)
No. of fusion segments	1.5 ± 0.7
One-level	27
Two-levels	14
Three-levels	3
Four-levels	1
No. of fusion spine levels	68
L1–2 (% of patients)	3 (4.4%)
L2–3 (% of patients)	9 (13.2%)
L3–4 (% of patients)	22 (32.4%)
L4–5 (% of patients)	34 (50.0%)
Blood loss (*ml*)	55.5 ± 60.1
Time in operating room (*min*)	116.5 ± 41.0
Length of stay (*days*)	16.4 + 4.4

All values are in mean ± standard deviation

Three patients underwent fusion at L1–2, 9 patients at L2–3, 22 patients at L3–4 and 34 patients at L4–5. The operation times ranged from 54 to 224 min (mean, 116.5 ± 41.0 min). Operative blood loss ranged from 4 to 342 ml (mean, 55.5 ± 60.1 ml). The length of hospitalization ranged from 9 to 28 days (mean, 16.4 ± 4.4 days). There was no case needing reoperation for inadequate decompression among the patients included in this study.

Comparison of patient background between the two groups showed that age, sex, number of fusion segments, and number of spine levels did not differ between the two groups. Further evaluation of surgical invasiveness showed that hemoglobin (Hb) level, estimated blood loss, and length of stay did not differ between the SP and DP groups. However, the operation time was approximately 31 min longer for the DP group (129.7 ± 36.0 min) than for the SP group (98.4 ± 41.3 min, *P* < 0.01) (Table 2).

**Table 2:** Comparison of two groups

Characteristic	DP group	SP group	*p*-value
No. of patients	26	19	–
Age *(years)*	70.9 ± 8.1	72.1 ± 8.5	0.679
Sex *(M, F)*	18: 8	11: 8	0.438
No. of fusion segments	1.6 ± 0.8	1.4 ± 0.7	0.412
One-level	14	13	
Two-levels	10	4	
Three-levels	1	2	
Four-levels	1	0	
No. of fusion spine levels	41	27	0.083
L1–2 No. of fusion spine levels	2	1	
L2–3 No. of fusion spine levels	7	2	
L3–4 No. of fusion spine levels	15	7	
L4–5 No. of fusion spine levels	17	17	
Blood loss *(ml)*	65.3 ± 70.2	42.2 ± 40.5	0.144
Pre-ope Hb *(g/dl)*	13.6 ± 1.8	14.0 ± 1.1	0.441
First-post-ope Hb *(g/dl)*	11.7 ± 2.0	12.1 ± 1.7	0.414
Change in Hb preope to first post ope *(g/dl)*	−1.9 ± 1.0	−1.9 ± 1.1	0.982
Time in operating room *(min)*	129.7 + 36.0	98.4 + 41.3	< 0.01
Length of stay *(days)*	16.6 + 4.4	16.2 + 4.6	0.835

All values are in mean ± standard deviation

*DP* dual position, *SP* single position

The height of the XLIF cage was 8–11 mm (8 mm, fifteen patients; 9 mm, nine patients; 10 mm, fifteen patients; 11 mm, two patients) for the DP group and 8–11 mm (8 mm, six patients; 9 mm, eleven patients; 10 mm, eight patients; 11 mm, two patients) for the SP group. All cages used were placed with 10° of lordosis. There were no statistically significant differences in cage height between the two groups (Table 3). When the placement of the XLIF cages was analyzed, there were no statistically significant differences between the two groups (DP group, 40.8 ± 10.5%; SP group, 41.3 ± 12.3%, *P* = 0.759).

**Table 3:** Implant dimensions for XLIF between two groups

	XLIF Implant Characteristics			
Height *(mm)*	8	9	10	11,12
No. of fusion segments (DP:SP)	21(15:6)	20(9:11)	23(15:8)	4(2:2)
Length (lateral) *(mm)*	45	50	55	60
No. of fusion segments (DP:SP)	5(4:1)	19(9:10)	38(24:14)	6(4:2)
Width (AP) *(mm)*	18	22		
No. of fusion segments (DP:SP)	68(41:27)	0(0:0)		
Lordosis *(°)*	0	10	15	
No. of fusion segments (DP:SP)	0(0:0)	68(41:27)	0(0:0)	

*DP* dual position, *SP* single position

Regarding pre- and postoperative image measurements, the segmental disc angle (SDA) was significantly increased after surgery in both groups. The preoperative SDA at the treated level averaged 3.0° and 4.1° for the DP and SP groups, respectively (*P* = 0.129) and was increased slightly postoperatively in both groups to 5.4° and 6.8°, respectively (*P* = 0.179), but did not differ significantly between groups. Similar results were obtained for lumbar lordosis (LL; L1–S1) and slipping length (SL), and there were no differences in LL and SL before and after surgery. For SL, significant postoperative restoration was observed in both groups. Average preoperative DH (Av DH) in both groups was also similar at baseline, (5.3 mm and 7.9 mm for the DP and SP groups), with an immediate significant postoperative correction to 10.0 mm and 11.3 mm, respectively. There was no significant difference in Av DH obtained between the two groups (4.6 mm and 3.5 mm for the DP and SP groups) (Table 4).

**Table 4:** Radiographic measures- LL, SDA, SL, and DH changes evaluated on pre-and postoperative X-ray

Characteristic	Assessment time	DP group	SP group	*p*-value
LL *(°)*	Pre	27.6 ± 13.2	28.2 ± 10.8	0.800
	Post (2 M)	30.1 ± 12.5	27.5 ± 9.5	0.522
	Pre→ 2 M change	2.6 ± 6.0	−0.7 ± 6.2	0.115
SDA *(°)*	Pre	3.0 ± 4.1	4.1 ± 4.8	0.129
	Post (2 M)	5.4 ± 4.0	6.8 ± 4.1	0.179
	Pre→ 2 M change	2.4 ± 4.3	2.7 ± 3.4	0.710
SL *(mm)*	Pre	0.9 ± 3.8	1.4 ± 3.7	0.669
	Post (2 M)	0.4 ± 2.9	0.5 ± 2.4	0.648
	Pre→ 2 M change	0.3 ± 1.8	0.8 ± 1.9	0.078
ADH *(mm)*	Pre	6.8 ± 4.0	9.7 ± 3.5	< 0.01
	Post (2 M)	12.2 ± 2.5	13.9 ± 2.5	< 0.05
	Pre→ 2 M change	5.8 ± 3.3	4.2 ± 3.5	0.098
PDH *(mm)*	Pre	4.8 ± 3.1	5.7 ± 2.0	0.093
	Post (2 M)	7.7 ± 2.3	8.7 ± 1.7	0.068
	Pre→ 2 M change	3.4 ± 2.1	2.8 ± 2.3	0.153
Av DH *(mm)*	Pre	5.3 ± 3.2	7.9 ± 2.4	< 0.01
	Post (2 M)	10.0 ± 1.9	11.3 ± 1.7	< 0.01
	Pre→ 2 M change	4.6 ± 2.4	3.5 ± 2.5	0.067

All values are in mean ± standard deviation

*LL* Lumbar lordosis, *SDA* Segmental Disc Angle, *SL* Slipping length, *ADH* Anterior Disc height, *PDH* Posterior Disc height, *Av DH* Average Disc height, *DP* dual position, *SP* single position

**Fig. 2:**
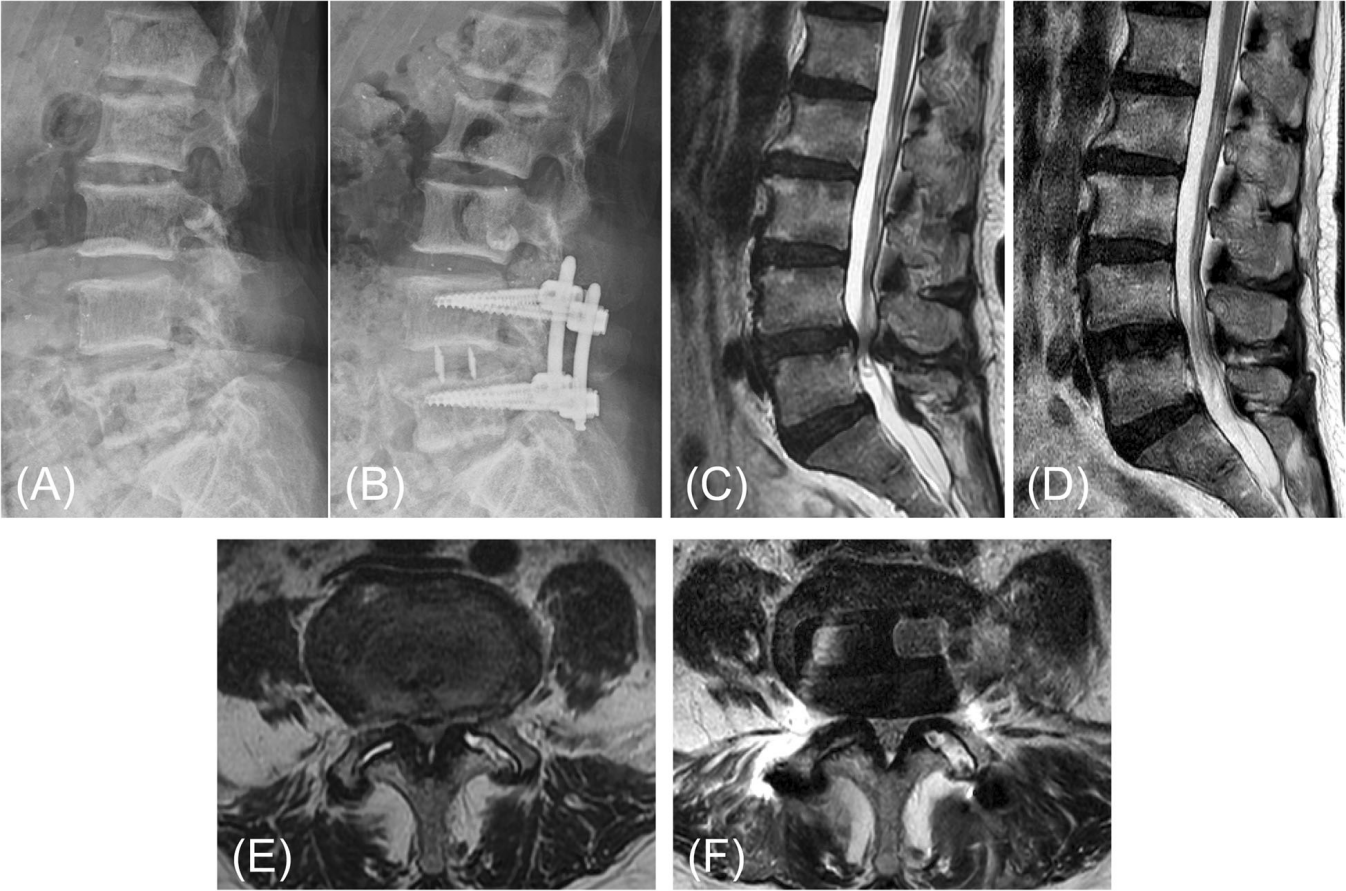
L4 degenerative spondylolisthesis with lumbar canal stenosis in a 65-year-old female (patient No. 15 in Table 1). **a** Lateral radiograph before surgery. **b** Lateral radiograph 2 months after surgery (XLIF plus SP-PPS in lateral decubitus position). Sagittal and axial magnetic resonance imaging before surgery (**c**, **e**) and 2 weeks after surgery (**d**, **f**). An increase in cross-sectional spinal canal area (CSA) (from 58.1 mm^2^ to 66.8 mm^2^) and diameter (from 4.2 mm to 9.1 mm) were observed after surgery, but the change was small at 2 weeks after surgery. However, the patient’s preoperative symptoms have improved, and she is progressing satisfactorily after the surgery

To evaluate indirect decompression, the CSA and the diameter of the dural sac were measured using MRI. As shown in Fig. 2, indirect decompression by LLIF for DS could enlarge the spinal canal after surgery. The results showed that CSA of the dural sac was significantly increased after surgery in both groups (DP group, from 55.3 to 78.4 mm^2^; SP group, from 54.7 to 77.2 mm^2^). We found an average increase in the CSA of the dural sac (21.9 and 22.6 mm^2^ for the DP and SP groups, respectively; *P* = 0.684), 42 and 41% increase, respectively from preoperative values. Central canal diameter also increased from 5.9 mm to 7.9 mm in the DP group and from 5.6 mm to 7.7 mm in the SP group. Statistically, there was no difference in the improvement rate of CSA (⊿ CSA) and diameter (⊿ Diameter) between the two groups. Detailed central canal area and diameter measurements are shown in Table 5.

**Table 5:** Radiographic measures- Canal dimension (CSA and diameter of dural sac) changes evaluated on pre-and postoperative MRI

Characteristic	Assessment time	DP group	SP group	*p*-value
CSA *(mm*^*2*^*)*	Pre	55.3 ± 34.3	54.7 ± 21.1	0.463
	Post (2 wk)	78.4 ± 38.1	77.2 ± 41.1	0.764
	Pre→ 2 wk change	21.9 ± 23.2	22.6 ± 28.1	0.684
Diameter *(mm)*	Pre	5.9 ± 2.6	5.6 ± 2.4	0.990
	Post (2 wk)	7.9 ± 2.4	7.7 ± 2.1	0.826
	Pre→ 2 wk change	2.0 ± 2.2	2.1 ± 2.0	0.925

All values are in mean ± standard deviation

*DP* dual position, *SP* single position, *CSA* cross-sectional spinal canal area

Analysis of the correlation between ⊿ CSA and cage position of the 68 levels showed a positive correlation (*r* = 0.448, *P* < 0.001). The correlation between ⊿ Diameter and cage position (*r* = 0.409, *P* < 0.01) was also found. However, there was no correlation between cage height and ⊿ CSA or ⊿ Diameter (Table 6).

**Table 6:** Spearman correlations mean (Spearman’s *r*) between ⊿CSA or ⊿diameter and cage parameters

	⊿CSA	⊿Diameter	Cage Position	Cage Height
⊿CSA	1.000	0.375**	0.448***	−0.101
⊿Diameter	0.375**	1.000	0.409**	0.135
Cage Position	0.448***	0.409**	1.000	0.035
Cage Height	−0.101	0.135	0.035	1.000

*CSA* cross-sectional spinal canal area

** *p*< 0.01, ***< 0.001 indicates significant differences between groups

## Discussion

We evaluated whether the perioperative and radiographic outcomes differed between SP and DP surgery. In the present study, indirect decompression of anterior–posterior spinal fusion with LLIF plus PPS fixation in the lateral decubitus position was evaluated by radiography and MRI for patients with DS. For the first time, it was revealed that similar levels of indirect decompression and disc angle could be obtained from the X-ray and MRI evaluations when PPS was inserted in the lateral decubitus position, as in the prone position. It has been thought that adequate restoration of lordosis could only be obtained in a prone position. However, the present study suggests that similar central canal dimensions and disc angles can be obtained without postural change after an LLIF procedure.

In this study, a significant increase in the central canal dimensions was observed in both groups after LLIF, but the change was smaller at 2 weeks after surgery. Previous studies of indirect decompression by LLIF have demonstrated that increases in the intervertebral disc and foraminal heights and the unbuckling of ligamentous structures can indirectly decompress neural elements. However, radiographs of indirect decompression of central canal stenosis using LLIF are less consistent [4, 14]. Oliveira et al. demonstrated an increase in average intervertebral disc height (42%) and central canal diameter (33%) at 43 lumbar levels [4]. It has been reported that the CSA of the dural sac will gradually expand over time. Thus, Ohtori et al. showed that the stability of the spine may induce changes in the lumbar ligamentum flavum and remodelling of the spinal canal [10]. Moreover, Elowitz et al. also found an improvement in clinical outcome scores even in patients with only a modest increase in spinal canal area after LLIF [2]. In support of these data, there have been a very interesting prospective study recently, the report showed that the thickness of the ligamentum fluvum and disc bulging decreased steadily over time after fixation, and cauda equina was visible in a majority of cases at 2 years after surgery [15]. The need for additional direct decompression after LLIF is a major topic that is frequently discussed [16, 17]. Some findings indicate that because severe central canal stenosis may reach beyond the limits of LLIF, it is necessary to investigate cases of severe stenosis. In our study, although the follow-up period was short, no patient required a secondary direct decompression after LLIF.

It is important to focus on the correction of anterior slippage. It has been suggested that slip correction could be achieved by inserting PPS in the lateral decubitus position; however, because the number of reported cases is small, further investigation of this issue is warranted.

It has been reported that the disc angles that can be obtained indicate that cage placement is also important for indirect decompression [6, 18, 19]; i.e., anterior placement is effective for the correction of spinal alignment, whereas central placement is advantageous for indirect decompression. The analysis in this study indicated that there was no difference in the cage position of the two groups and no correlation with the disc angle that was obtained. In addition, the cage position correlated with ⊿CSA and ⊿Diameter but not with the cage height, suggesting that the cage position might be more important for indirect decompression than the cage height.

In our previous report, we found that SP-PPS technique can shorten the operation time compared with DP-PPS technique. The results showed that the operation time of SP surgery was about 32 min shorter than that of DP surgery [20]. A recent study evaluating the costs of LLIF estimated that the operating room cost is approximately $83/min [21]. Although such evaluations are not straightforward, the reduced operating time could result in a potential saving of $2656 per case. Most of this time difference is probably related to the need during DP surgery to change the patient’s position, re-prepare, and re-drape when transitioning from a lateral decubitus position to a prone position. Another possible reason is the use of a guidewire-less PPS system. The insertion of conventional PPS requires several steps including a needle, a guidewire, a tap, and a screwdriver. Therefore, inserting PPS in the lateral decubitus position can be complicated and the working space is narrow; there is also a short learning curve for using fluoroscopy when inserting downside PPS. In contrast, the newly developed PPS system (VIPER PRIME™) that we used is characterized by the fact that these processes can be performed in one step.

This study had important limitations that stemmed from its retrospective, single-cohort design and the limited postoperative observation period. Another limitation is that the sample size was small. However, to our knowledge, this is the first report of fixation of PPS after LLIF surgery without changing the patient’s position that compares indirect decompression using MRI. The effect of the small sample size was taken into consideration, but the SP procedure also resulted in a similar decompression of the spinal canal to that achieved in the prone position, and the operation time was significantly reduced. A final limitation of this study is the lack of information about the accuracy of insertion of the lateral PPS. We believe that it is important to demonstrate the equivalence of SP and DP surgery and whether PPS placement can be performed accurately in the decubitus position. However, we could not compare CT before and after surgery because we did not have a full set of CTs before surgery or early after surgery for all patients. To perform CT in all patients without symptoms after surgery, informed consent for a prospective study is necessary. Further studies are required to clarify these issues.

## Conclusions

This study compared the perioperative characteristics and image evaluation of patients after LLIF, with 19 patients undergoing SP surgery and 26 DP surgery. In SP surgery, the entire lumbar surgery, including approach, discectomy, interbody fusion, and percutaneous screw insertion, was performed in the lateral decubitus position. Interestingly, the spinal canal enlargement and disc angle obtained after the surgery did not differ between SP and DP surgery, and SP surgery could reduce the average surgery time by about 31 min. Overall, this indicates that SP surgery after LLIF is likely to be a very useful technique to both the surgeon and patient.

## Data Availability

Data available upon request from corresponding author.

## Abbreviations

CSA: Cross-sectional area
CT: Computed tomography
DH: Disc height (DH)
DP: Dual-position surgery
DS: Degenerative spondylolisthesis
Hb: Hemoglobin
LLIF: Lateral lumbar interbody fusion
MRI: Magnetic resonance imaging
PPS: Percutaneous pedicle screw
SP: Single-position
XLIF: Extreme lateral interbody fusion
